# Research Progress on the Mechanism of Itaconate Regulating Macrophage Immunometabolism

**DOI:** 10.3389/fimmu.2022.937247

**Published:** 2022-06-23

**Authors:** Jia Shi, Cheng Cai

**Affiliations:** Department of Neonatology, Shanghai Children’s Hospital, School of Medicine, Shanghai Jiaotong University, Shanghai, China

**Keywords:** immunometabolism, itaconate, tricarboxylic acid cycle, research progress, macrophages

## Abstract

The field of immunology is undergoing rapid and dramatic changes. Immunometabolism, a change in metabolic pathways within immune cells, is a key determinant in the activation of immune cells, and intermediates of immunometabolic processes which can influence inflammatory gene expression and play a role in inflammation. Itaconate is one of the most representative metabolites, produced in the tricarboxylic acid cycle (TCA cycle), which links macrophage metabolism, oxidative stress response and immune response to regulate macrophage activity, playing an important role in the function of macrophages. In this paper, we review the mechanisms of the metabolite itaconate and its derivatives in the regulation of macrophage immune metabolism, intending to gain further insight into the role and mechanisms of this metabolite in macrophages and provide new ideas for the mechanisms and treatment of clinical diseases.

## Introduction

Macrophage is a key component of innate immunity. When encountering signals associated with microbial infection or tissue damage such as lipopolysaccharide (LPS) and interferon-γ (IFN-γ), M0 macrophages rapidly polarize to the classic M1 phenotype. M1 activation is characterized by increased production of pro-inflammatory cytokines and nitric oxide (NO), rapid eradication of invading pathogens. Repeated or prolonged exposure to a certain stimulus is followed by more M2-like phenotypes, decreased production of certain pro-inflammatory cytokines, and increased production of anti-inflammatory cytokines to prevent cell damage and promote tissue repair (1). Macrophages are stimulated by different environments around them to differentiate and form different subtypes, such as M1 macrophages and M2 macrophages This change between macrophage subtypes is macrophage polarization.

The correct macrophage phenotype status is essential for innate immune function. Dysregulation of this process is associated with both autoimmune and sepsis diseases. There is growing evidence that shifts in different functional states of macrophages are accompanied by and dependent on metabolic reprogramming. This reprogramming provides the cells with the energy and metabolites they need to function and regulates metabolite levels. Recent studies have highlighted the importance of the tricarboxylic acid cycle (TCA cycle) in M1 macrophage activation, which is associated with the rapid accumulation of itaconate and succinic acid and changes in the redox state of cells (2). Evidence also suggests that these metabolites have important immunomodulatory effects, and changes in their levels can affect the inflammatory state of macrophages (3).

The TCA cycle, also known as the citric acid cycle or the tricarboxylic acid cycle, is the core biochemical process of eukaryotes. Advances in immunometabolism have upended immunologists’ understanding of the cycle, arguing that the TCA cycle has become the immune metabolic hub of macrophages (4). Many TCA cycle metabolites play a role in controlling monocyte phenotype and effector function, and specific macrophage activation or polarization states can in turn affect the corresponding metabolites (5). Itaconate is an immunomodulatory derivative of TCA cycle, produced from TCA cycle and highly expressed in activated macrophages, it can cause changes in metabolites and mitochondrial respiration in macrophages, and changes in metabolic state will follow by changes in cell function, such as polarization of macrophages and increased release of cytokines. This review will discuss the main evidence for the function of itaconate and its derivatives in inflammation, and through the study of these metabolites acting on corresponding signaling molecules and activated signaling pathways, it is hoped to provide more therapeutic opportunities for related diseases.

### History of the Development of Itaconate

In 2011,Strelko et al. (6) in Massachusetts, USA revealed that itaconate is generated by the TCA cycle intermediate product being catalyzed decarboxylation, and the intermediate metabolite citric acid is converted to cis-aconitic acid under the catalysis of cis-aconitase, which in turn undergoes decarboxylate reaction and produces itaconate under the action of cis-aconitate decarboxylase (*CAD*, also known as aconitate decarboxylase 1, *ACOD1*) *(* 7). In 2013, Michelucci et al (8) discovered in activated macrophages an immunoresponsive gene 1 protein (IRG1) that can be induced to be expressed by lipopolysaccharide (LPS), which is highly expressed in mammalian inflammatory macrophages. *IRG1* encodes the enzyme *CAD*, which catalyzes the TCA cycle’s decarboxylation reaction of cis-aconitic acid to form itaconate, revealing the itaconate manufacturing route in macrophages at a different level (8).

In 2016, Lampropoulou et al. first discovered that itaconate has anti-inflammatory effects (9). Subsequent studies have also suggested that itaconate can exert an anti-inflammatory effect in LPS-stimulated activated macrophages, and is one of the metabolites with the highest inducibility rate in activated macrophages. In mouse bone marrow-derived macrophage (BMDM), knockout of the *IRG1* gene prevented itaconate synthesis and secretion by either LPS or IFN-γ. *IRG1* gene silencing results in a significant decrease in intracellular itaconate levels and a significant decrease in antimicrobial activity during bacterial infection (10). These studies suggest that IRG1 encoding the key enzyme for itaconate synthesis can participate in the regulation of the inflammatory response by altering its itaconate levels within cells, linking cellular metabolism to immune defenses by catalyzing the production of itaconate. The development of itaconate and its derivatives has great potential in the treatment of inflammatory diseases (11), and IRG1 and itaconate are becoming new targets for disease treatment.

### Derivatives of Itaconate

Itaconate is an α,β-unsaturated carboxylic acid with strong polarity, which can alkylated protein cysteine residues through the Michael addition reaction to form a 2,3-dicarboxypropyl adduct (12). itaconate is structurally and chemically similar to other metabolites such as phosphoenol-type pyruvate, succinic acid, malonic acid, and fumaric acid. itaconate does not easily penetrate cell membranes, and after mitochondrial synthesis, it needs to enter the cytoplasm to exert anti-inflammatory effects. To overcome the disadvantage that itaconate is not easy to pass through cell membranes, the researchers synthesized dimethyl itaconate (DI) and 4-octyl itaconate (4-OI), two membrane-permeable itaconate derivatives that can pass through the cell membrane directly into the cell without transporters and convert into itaconate within the cell (13).

The lack of negative charge on the conjugated ester group in DI increases its reactivity to Michael addition, making it a far superior Nrf2 activator to itaconate, similar to the potent Nrf2 activator dimethyl fumarate (DMF) (14). Itaconate derivative 4-OI can be hydrolyzed into itaconate by cytoplasmic lipase after entering the cell, which can also exert anti-inflammatory effects (15). DI degrades rapidly within cells and cannot be converted to endogenous itaconate (16). The structure of 4-OI determines that its thiol reactivity and its electrophilicity are lower than that of itaconate, so it is more suitable for the study of endogenous itaconate (14). However, after the itaconate derivatives enter the body, the structural changes may produce some additional effects that are not related to itaconate, so the role of endogenous itaconate in the body cannot be fully simulated. Despite the above, itaconate derivatives still provide a viable pathway for studying the role of itaconate in macrophage function, requiring more in-depth research by researchers.

Itaconate regulation of macrophage immune metabolism can be achieved through a variety of pathways, the main pathways being Kelch-like epichlorohydrin (ECH) associated protein 1 (Keap1)/nuclear factor erythroid related factor 2(Nrf2), Nrf2 regulated antioxidants by alkylated residues of Keap1 (17). Recently, there have been new ideas that metabolites such as itaconate can regulate macrophage activity through other pathways, such as the stimulator of interferon genes (STING) pathway, ferroptosis, etc., which have revealed that itaconate is a powerful anti-inflammatory metabolic derivative with potential clinical applications.

## Itaconate Activates Keap1/Nrf2 to Regulate the Immune Function of Macrophages

**Figure 1:**
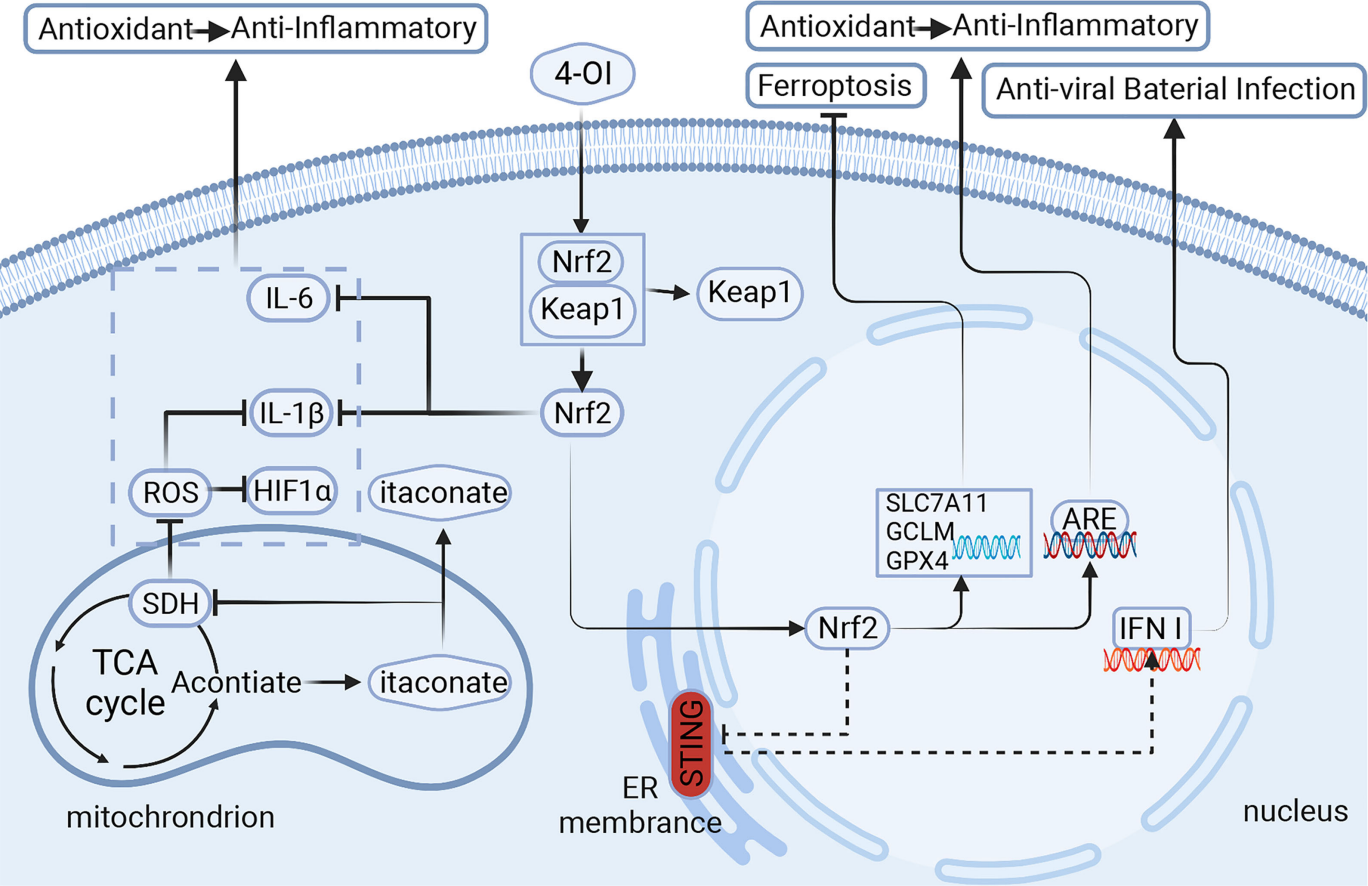
Itaconic is derived from TCA cycle metabolites and is highly expressed in macrophages. It regulates the metabolites in macrophages through different signaling pathways, which affect the polarization of macrophages and the release of cytokines, and then plays a role *in vivo*. Signaling pathways of itaconate and its derivatives:1.Itaconate activates Nrf2 for anti-inflammatory and antioxidant. 2.Itaconate negatively regulates STING for anti-viral and bacterial infection. 3.Itaconate inhibits ferroptosis in macrophages. 4-OI,4-octyl itaconate;Nrf2,nuclear factor erythroid related factor 2; Keap1,Kelch-like ECH-associated protein 1; ARE, antioxidant response element; STING, stimulator of interferon genes; ROS, reactive oxygen species; SDH, succinate dehydrogenase; HIF1α,hypoxia inducible factor 1 subunit α; GPX4/SLC7A11/GCLM, markers of ferroptosis.Created with BioRender.com.

The Keap1/Nrf2 signaling pathway is a redox reaction system that regulates 1%-10% of the body’s genes, which mainly encode detoxification and redox defense functions, maintaining cellular redox balance and metabolism (18). Nrf2 belongs to the transcription factor Cap-n-collar(CNC) family, consisting of 605 amino acids divided into seven highly conserved functional domains called Neh1-Neh7 (19). Under normal physiological conditions, Nrf2 is localized in the cytoplasm and binds to Keap1, which degrades the Nrf2 proteasome through the Cullin3 ubiquitin complex, exerting an inhibitory effect on Nrf2 (20); and once Nrf2 is activated, the Keap1 cysteine residue undergoes alkylation, and Nrf2 is isolated from Keap1, translocated to the nucleus and bound to the antioxidant response element (ARE), activates transcription of Nrf2-dependent genes associated with anti-inflammatory and antioxidant-related genes, promotes the expression of a variety of antioxidant and anti-inflammatory proteins, and thus exerts a cytoprotective effect (21) (**Figure 1**).

Oxidative stress leads to oxidative damage to biomolecules, causing damage-associated molecular patterns (DAMPs) production and cytokine release *in vivo*. Cytokines can activate signaling pathways downstream of pattern recognition receptors (PRRs), such as nuclear factors κB (NF-κB), JAK, STAT, and MAPK, resulting in increased release of cytokines and chemokines, recruiting and activating more inflammatory cells, and causing a systemic chronic inflammatory response. Asthma is a complex inflammatory disease characterized by inflammation of the airways and hyperreactivity. Recent studies have suggested that difficulties in treating severe asthma are closely related to oxidative stress, and that the combination of anti-inflammatory and antioxidant stress helps in the treatment of severe asthma (22). In the same way, in the acute inflammatory phase of sepsis, the imbalance of the redox state changes to a pro-oxidation state, and correspondingly, the function of various endothelial cells changes under oxidative stress, progressing to pro-inflammatory, procoagulant and pro-adhesion phenotypes (23). Therefore, the body’s inflammatory state is closely related to the oxidative stress state, the inflammatory response and oxidative stress are almost twins. It’s not difficult to see why Keap1-Nrf2 is so critical for regulating the inflammatory response as an important mechanism for oxidative stress.

Itaconate has recently been recognized as a regulator of macrophage function (24, 25). Studies have shown that Nrf2 protein levels in wild-type macrophages activated by LPS are higher than in macrophages with the deletion of the *IRG1* gene, revealing that endogenous itaconate can induce Keap1/Nrf2 responses in macrophages. itaconate derivative 4-OI activates Nrf2 by Keap1 alkylation, inhibits certain pro-inflammatory cytokines in macrophages *in vitro*, and has a protective effect in LPS lethal models *in vivo* (26). Nrf2 can also activate the transcription of important macrophage function-related genes, such as macrophage receptors with collagen structures (MARCO), receptors needed for bacterial phagocytosis, differentiation cluster 36 (CD36), scavenger receptors that oxidized low-density lipoprotein(LDL), and viral monitoring mediator interleukin-17D (IL-17D) (26). 4-OI activates Nrf2 *via* alkylating the cysteine of Keap1, which may be part of the reason for its anti-inflammatory effects. It has also been proposed that 4-OI can induce glyceraldehyde-3-phosphate dehydrogenase (GAPDH) Cys 22 residue alkylation, affect GAPDH activity to inhibit glycolysis, and glycolysis reduction can hinder macrophage activation, reduce the production of pro-inflammatory cytokines and inhibit inflammation (27). In summary, Nrf2 can regulate the innate immunity of macrophages by promoting intra-macrophage metabolic reprogramming.

### *In Vitro* Validation of Itaconate Regulating Immune Metabolism of Macrophage by Nrf2/Keap1

Several studies have demonstrated at the cellular level that itaconate and their derivatives can exert anti-inflammatory and immunomodulatory effects by influencing the metabolic function of macrophages through the Nrf2/Keap1 pathway. A study conducted on RAW264.7 cell which is a macrophage cell line that was established from a tumor in a male mouse induced with the Abelson murine leukemia virus showed that 4-OI reduced the accumulation of neutrophils and secretion of inflammatory factors (P<0.05) in LPS-induced acute lung injury (ALI), and significantly reduced the active oxygen species content of tissues (P <0.05), simultaneously upregulating the expression of Nrf-2 and Nrf-2 target genes in lung tissue (28).

The metabolomics of bone marrow-derived macrophages (BMDMs) produced from the *in vitro* development of bone marrow cells taken from wild-type (WT), Keap1 knockdown (Keap1 KD), and Nrf2 knockout (Nrf2 KO) mice have been examined.Nrf2 activation was found to significantly increase antioxidant metabolites, including GSH, taurine, β-alanine, and carnosine, while Nrf2 destruction significantly reduced levels of these metabolites. In addition, both activation and destruction of Nrf2 can lead to significant alterations in mitochondrial metabolites, such as those involved in fatty acid oxidation (FAO) (carnitine, palmitoyl carnitine, hexyl carnitine, and tetradecyl carnitine), TCA cycles (fumaric acid, malic acid, and 2-ketoglutaric acid), and bioenergy (NAD, creatine, creatine phosphate) (26). In stimulating these three types of cells with LPS, the antioxidant response and bioenergy-related metabolites increase significantly, and the abundance of several amino acids changes to varying degrees (26). These experimental data show that the derivative of itaconate, 4-OI, activates Keap1/Nrf2 to reduce oxidative stress to fight inflammation. At the same time,the occurrence of metabolic pathways and metabolites such as antioxidants, lipid oxidation and TCA cycle in macrophages has certain changes, thereby regulating immune function to play a protective role on the body. Therefore, it is proved at the cellular level that it affects macrophage immune function through Nrf2/Keap1.

### *In Vivo* Validation of Itaconate Regulating Immune Metabolism of Macrophage *via* Nrf2/Keap1

In a mouse methicillin-resistant Staphylococcus aureus (MRSA) model of pneumonia, by increasing Nrf2, 4-OI decreased the expression of inflammatory factors in macrophages and caused a persistent anti-inflammatory impact by binding to Keap1 vesicles. Mice with Nrf2 inhibitor (ML385) and Nrf2 knockout eliminated the protective effect of 4-OI on MRSA-induced inflammation both *in vitro* and *in vivo* (29). *In vitro*,4-OI inhibits the inflammatory response and oxidative stress damage induced by Lipoteichoic acid (LTA) in RAW264.7 cells, and Nrf2 inhibitors (ML385) can eliminate the anti-inflammatory effects of 4-OI in LTA-induced macrophages (29). The study demonstrated in animal models that the effect of itaconate on macrophage function can be performed *via* the Nrf2/Keap1 pathway, and then further validated in cell line experiments.

Keap1 is the primary negative regulator of Nrf2, and another mouse study found that downregulation of the Keap1 gene, as well as activation of Nrf2 to pharmacological levels, alter lipid metabolism, leading to upregulation of carboxylesterase 1 (Ces1) and acyl-CoA oxidase 2 (acyl-CoA oxidase 2, Acox2), lowering triglyceride levels (30). These studies have further validated *in vivo* that itaconate influences metabolic pathways through the Keap1/Nrf2 pathway, reduces inflammation-induced organ damage, and regulates macrophage immune function. In summary, itaconate is located at the center of macrophage metabolism, and an in-depth study of the mechanism of itaconate’s action *in vivo* can help us better understand the biology of Nrf2 and its regulatory role in innate immunity.

## Itaconate Negatively Regulates the STING Pathway to Modulate Macrophage Immune Metabolism

STING is a key receptor protein in the natural immune response pathway (31). After PRRs recognize cytoplasmic viral nucleic acids, the conformation changes after STING binds to these receptors. STING uses a vesicle transport system to transport the endoplasmic reticulum to the Golgi apparatus. Tank-binding kinase 1 (TBK1 kinase) and interferon regulatory factor 3 (IRF3) transcription factors were recruited in the Golgi apparatus to promote phosphorylation and dimer formation of IRF3. IRF3 is transferred to the nucleus, inducing the expression of interferon type I (IFN I) and inflammatory factors, activating the natural immune response (32, 33).

STING activation can be divided into cyclic GMP-AMP synthase (cGAS) dependent and non-cGAS-dependent forms. The most popular recent pathway is the cGAS-STING pathway, which consists mainly of cGAS and STING, activating the downstream IFN response to protect the host from viral infection. After sensing viral double-stranded DNA, cGAS catalyzes the formation of ATP and GTP into 2’,3’ cyclized GMP-AMP (2’,3’-cGAMP) (also known as “non-classical” cGAMP), a cyclic dinucleotides (CDNs) that act as a second messenger to bind to the binding protein STING, a localized endoplasmic omentum (34), by promoting IFN and hundreds of IFN-stimulating genes (interferon The expression of stimulator genes, ISGs), triggers an antiviral immune response (35) and metabolic reprogramming of macrophages (36).

The STING pathway can be both induced and inhibited and is a promising medical target in areas such as cancer, antiviral, anti-inflammatory response, and vaccine development (37). Recent studies have also revealed that STING is a universal mechanism within the biological world, and all microorganisms that can carry DNA into the host cytoplasm, such as DNA viruses, bacteria, parasites (such as malaria) and retroviruses (such as HIV), may trigger the STING pathway (38) (**Figure 1**).

### Nrf2, a Negative Regulator of STING-Dependent Type I Interferon Reactions

IFN I (IFN-α and IFN-β) are at the heart of immune protection against viral infections, and the production of IFNα/β relies on the inherent recognition of cytoplasmic viral nucleic acids by PRR, including the DNA sensor cGAS (39), which signals through the adapter STING. Studies have revealed that 4-OI can significantly reduce IFN I-reactive protein. Experimental results show that when compared with wild-type (WT), LPS-induced BMDMs are pretreated with 4-OI for 3 h in advance before being knocked down by Keap1, the protein level of IFN-β in cells is reduced, whereas it is elevated in cells that silence Nrf2. qPCR analysis further showed that the effect of Nrf2 on IFN-β occurred at the transcriptional level. Compared with WT cells, IFN-β target interferon-inducible protein (*IFIT2*) increased in Nrf2-silenced cells and reduced expression in KEAP1 knockdown cells. NAD(P)H: quinone oxidase-1 (NQO1) and C-X-C motif chemokine 10 (CXCL10) are downstream targets of the IFN I signaling pathway. Murine RAW 264.7 macrophage-like monocytes treated with the pharmacologic Nrf2 activators the isothiocyanate sulforaphane or the pentacyclic cyanoenone CDDO increased levels of NQO1 mRNA and decreased levels of the chemokine CXCL10 mRNA (26). These findings revealed that Nrf2 activation inhibits IFNβ and *IFIT2*, supporting the association between the anti-inflammatory effect of Nrf2 and the inhibition of the IFN I response.

The researchers evaluated the entire transcriptome of the human epithelial cell line A549 cells, then used functional clustering analysis to compare the differential genes found in the control and siRNA-silenced Nrf2 groups. The pathways of antiviral and IFN I responses in the siRNA-silencing Nrf2 group were significantly upregulated, and the *TMEM173* gene which encodes STING, a protein involved in the IFN I response and antiviral signal transduction, had variable levels of expression (40). STING is at the heart of the downstream signal release of the IFN I response to cytoplasmic DNA and cytoplasmic DNA sensor cGAS, which is involved in the IFN I response as well as antiviral sensing and signaling. These data suggest that STING’s antiviral effect is partly exerted through IFN I and that there is an intrinsic link between the inhibitory effect of Nrf2 on the IFN I response and the STING pathway. The researchers believe that STING is the link between the metabolite itaconate and the antiviral nucleic acid-sensing, thus linking the two to work together to regulate the body’s function.

### Itaconate-Mediated STING Suppression Achieved by Nrf2

4-OI is a potent Nrf2 activator, when the Nrf2 promoter is stimulated by 4-OI, its activity increases in a dose-dependent manner. In cells of the human monocyte line THP1, a significant decrease in *TMEM173* mRNA levels was observed after stimulation with 4-OI, proving that the itaconate/Nrf2 chain plays a role in the regulation of STING, which can be negatively regulated by itaconate *via* Nrf2. 4-OI treatment significantly impairs STING protein expression and STING-dependent signaling for cGAMP, while also inhibiting the release of type I IFN, downstream STAT1 signaling, and subsequent ISG induction. To make the findings more compelling, the researchers conducted a number of validation studies in different cell lines (40). The silencing of Nrf2 by siRNAs in this study reduced the inhibition of STING in HaCat cells so we came to the conclusion that the effect of 4-OI treatment depended on Nrf2. It was further concluded that itaconate negatively modulated STING-dependent IFN I *in vivo* through the Nrf2 signaling pathway.

Metabolic reprogramming in immune cells such as macrophages or dendritic cells can be induced by LPS stimulation (41). It is well known that LPS can activate Nrf2. Treatment of PMDC05 cells (human plasma cell-like denritic cell line) with the TLR4 agonist LPS or TLR7 agonist Gardiquimod leads to increased lactic acid release, increased glucose consumption, increased itaconate accumulation, and increased expression of HIF1α, a marker of metabolic reprogramming of innate immune cells. After treatment with TLR4 or TLR7 agonists, the expression of the Nrf2-induced enzyme heme oxygenase-1 (HO-1) is enhanced. These studies demonstrate a possible connection between Nrf2 activation and STING inhibition in the context of LPS-induced metabolic reprogramming (40).

These studies reveal that itaconate and its derivative 4-OI inhibit the expression of STING through Nrf2 while supporting the close link between antiviral immunity and metabolism. 4-OI treatment severely impairs STING protein expression and STING-dependent signaling to cGAMP, suggesting that there may be a link between Nrf2 activation and STING inhibition during LPS-induced metabolic reprogramming.

Nuclear factor-kB (NF-κB) can also be activated by STING, and it has been found that phosphorylated IRF3 dimers are dimerized and translocated into the nucleus, and can also work with NF-kB to turn on the expression of inflammatory cytokines, leading to immune response (42).In a study of osteoarthritis(OA), exogenous supplementation with itaconate activated Nrf2 and inhibited the STING-dependent NF-κB pathway, thereby alleviating IL-1β-stimulated chondroblast inflammation, extracellular matrix (ECM) degeneration, and aging. Itaconate can also modulate the polarization of RAW264.7 macrophages, further reducing chondrocyte apoptosis (43).

The IFN-independent activity of STING found to be prevalent in macrophages and T cells can produce antiviral immunity, despite the fact that the IFN I response is commonly considered the major signaling function of STING (44, 45). These studies have raised new ideas for downstream pathways activated by itaconate negative regulation of STING, and STING has recently been shown to induce a non-normative autophagy pathway (46) that is not related to IFN and has antiviral activity against HSV-1 infection *in vitro* (47).

Itaconate’s immunomodulatory effect is a popular focus of research right now. This effect plays a role in regulating the immune metabolism of macrophages, and may be achieved in part by the inhibition of Nrf2 on STING. At present, there is still a lack of direct evidence that the metabolite itaconate acts on STING, and researchers need to conduct *in vitro* and *in vitro* experiments to verify these. However, these studies make connections between immune metabolism and nucleic acid perception, providing new ideas for the mechanism of action of macrophage inflammation, and STING inhibitors can also provide new directions for disease treatment.

## Itaconate Inhibits Ferroptosis to Play a Role in Macrophage Function

In ferroptosis, a non-apoptotic form of cell death in which iron-dependent regulatory necrosis caused by membrane damage mediated by massive lipid peroxidation differs from apoptosis, autophagy, or other forms of cell death (48). The essence of ferroptosis is that glutathione (GSH) depletes, and glutathione peroxidase 4 (GPX4) activity decreases (49). Due to this reaction, lipid oxides can’t be GPX4-catalyzed glutathione reductase reaction metabolism, after which divalent iron ion oxidizes lipids to produce reactive oxygen species, thereby promoting the occurrence of ferroptosis (50). Ferroptosis is precisely regulated at multiple levels, including epigenetic, transcriptional, post-transcriptional, and post-translational levels. There is increasing evidence to suggest that excessive or inadequate ferroptosis may be associated with physiological and pathophysiological processes and is accompanied by a dysregulated immune response (51).

### Effect of Itaconate on Ferroptosis by Inhibiting Lipid Peroxidation

The new study has found that there are significant differences in ferroptosis-related genes in sepsis-induced ALI mice pretreated with itaconate derivatives (52). Markers of ferroptosis are cystine/glutamate transporter (SLC7A11), GPX4, and prostaglandin-in-peroxide synthase 2 (PTGS2) (53). After 4-OI administration, the marker of ferroptosis, GPX4 increased significantly, while PTGS2 decreased (52). The presence of gene and protein changes associated with ferroptosis in experimental mice suggests itaconate may influence this process. Lipid peroxide is known to be a key process of ferroptosis, and the lipid peroxidation of GPX4 against ferroptosis is accomplished with the assistance of glutamate-cysteine ligase (GCL), a regulatory subunit of GCL (53). 4-OI can significantly increase GCLM levels and reverse LPS-induced decrease in GCLM levels. Previous studies have confirmed that it can be controlled by Nrf2 (52).

### Itaconate Affects Lipid-Dependent Ferroptosis Through Nrf2

Nrf2 is a major regulator of the antioxidant response, not only maintaining proper redox homeostasis, but has also been shown to play a key role in mediating other key metabolic pathways, including protein homeostasis, iron/heme metabolism, carbohydrate and lipid metabolism, and apoptosis. Dysregulation of the NRF2 pathway contributes to the development or progression of various pathologies (54). According to previous data, the oxidative stress effects of Nrf2/Keap1 modulate ferroptosis in sepsis mice (55, 56). Therefore, based on these studies we can speculate that the effect of itaconate on ferroptosis was achieved by modulating Nrf2 in LPS-induced ALI mice.

On the basis of this hypothesis, the researchers have conducted an in-depth discussion on the mechanism of itaconate’s role in ferroptosis and found that itaconate derivatives are dependent on Nrf2 to counter ferroptosis. Nrf2 can directly or indirectly regulate the expression of GPX4 and genes associated with GSH synthesis (57).In LPS-stimulated THP-1 cells, 4-OI pretreatment significantly increased the expression of Nrf2, GPX4, SLC7A11C, and GCLM, with an increased GSH and GSH/GSSG ratio and a decrease in MDA and ROS levels. By silencing Nrf2 with siRNA techniques, GPX4 levels in cells decreased significantly. Protein and mRNA levels of GCLM and SLC7A11 are reduced and cannot be reversed by 4-OI (52). This illustrates that in mice knocked out of Nrf2, the protective effect of 4-OI is almost completely gone. To further corroborate the above findings, the relevant trials were conducted again in Nrf2-KO mice to construct ALI models, the loss of Nrf2 aggravated the sepsis-induced ALI, and the protective effect of 4-OI on Nrf2-deficient ALI mice disappeared. The researchers found that 4-OI protects LPS mice by modulating Nrf2 against ferroptosis, while also reducing LPS-induced tissue iron levels in Nrf2-deficient mice, revealing that 4-OI can also regulate cytoplasmic iron through Nrf2’s non-dependent mechanism (52). Despite the above findings, it can still be concluded that itaconate inhibits ferroptosis in monocyte macrophages by activating Nrf2 and increasing Nrf2 levels, and has a protective effect on ALI where sepsis occurs. Multiple genes associated with iron metabolism and lipid peroxidation are regulated by Nrf2 transcription, so pharmacologically modulating the Nrf2 pathway to target upstream regulators of ferroptosis is one of the options for treating ferroptosis-related diseases.

### Effect of Inhibition of Ferroptosis by Itaconate on Macrophage Function

The regulation of ferroptosis by itaconate is accompanied by disorders of lipid peroxidation. Metabolic reprogramming refers to a change in metabolic state, and metabolic reprogramming of macrophages is inextricably linked with their polarization; therefore, some people have speculated that itaconate’s impact on ferroptosis would be partly dependent on macrophage polarization. Studies have shown that enrichment of inducible nitric oxide synthase (iNOS)/activated M1 macrophages/microglia can modulate susceptibility to ferroptosis (58). Because iNOS-dependent NO production is a negative regulator of iron apoptosis that promotes the survival of M1 macrophages, *in vivo*, M1 has a higher resistance to pharmacologically induced iron apoptosis compared to M2 macrophages. So GPX4 deletion induces iron apoptosis only in M2, not M1 macrophages (59).

Some researchers have also proposed the other view that the polarization of macrophages is related to the iron load during ferroptosis. Iron load promotes the polarization of M1 macrophages and induces the release of inflammatory factors such as IL-6, IL-1β, TNF-α within M1 macrophages (60). The effect of ferroptosis on macrophage function is multifaceted, it can not only affect the polarization of macrophages, but also initiate the aggregation of macrophages (61).Ferroptosis is present in a variety of diseases, and the removal of ferroptosis cells is performed by macrophages. Damaged-associated molecular pattern molecules (DAMPs) can recruit and activate macrophages. Ferroptosis-related cells can release a type of DAMP called High Mobility Group Box 1(HMGB1). HMGB1 promotes macrophage clearance of ferroptosis cells to mediate macrophage inflammation (62).

As a result, these studies have demonstrated a new role of itaconate in inhibiting ferroptosis *in vivo* and *in vivo*. In addition to exerting an anti-inflammatory effect, itaconate may inhibit host cell death, both of which may serve as protective mechanisms in the body, providing new insights into understanding the metabolic basis of ferroptosis and the potential application of itaconate in the treatment of inflammatory diseases. In future studies, the relationship between 4-OI and iron metabolism needs to be explored further.

## Conclusion

Advances in the field of cellular immune metabolism have led to a deeper understanding of the role of macrophages in the inflammatory infection process. Itaconate as a metabolic intermediate in the TCA cycle can alter the macrophage immune response in a variety of pathways and has the potential to significantly affect inflammatory outcomes. Itaconate acts on Nrf2/Keap1 to exert antioxidant and anti-inflammatory effects, negatively modulates STING pathways to play an antiviral and bacterial infection role, and inhibition of ferroptosis can play a role in lipid peroxidation-related degenerative diseases.

Itaconate has metabolic cell type specificity. Its production is almost exclusively confined to immune cells, including microglia. It can be used in endogenous form or derivatives, not only has a powerful anti-inflammatory effect, but also has a huge potential for immune metabolism. Studying the regulatory mechanism of itaconate and its alternative derivatives in the immune metabolism process of macrophages can provide new insights and ideas on the mechanism of action of macrophages in innate immunity; secondly, itaconate as a regulator of immune function, the therapeutic role in different diseases has been discovered, and metabolites can be considered as new interventions for clinical application in the future, and the complexity of these metabolic pathways and their derivatives may produce new treatment methods, which will have a significant impact on the pathogenesis of a variety of diseases. We can’t wait to further analyze this interesting metabolite as a key regulator of immunity and host defense.

## Author Contributions

All authors listed have made a substantial, direct, and intellectual contribution to the work, and approved it for publication.

## Conflict of Interest

The authors declare that the research was conducted in the absence of any commercial or financial relationships that could be construed as a potential conflict of interest.

## Publisher’s Note

All claims expressed in this article are solely those of the authors and do not necessarily represent those of their affiliated organizations, or those of the publisher, the editors and the reviewers. Any product that may be evaluated in this article, or claim that may be made by its manufacturer, is not guaranteed or endorsed by the publisher.
